# A carbon trail to follow: unveiling itaconate’s metabolism in vivo

**DOI:** 10.1097/IN9.0000000000000072

**Published:** 2025-10-27

**Authors:** Denis E. Anisov, Sally A. Clayton, Maxim A. Nosenko

**Affiliations:** 1 INSERM UMR-S1151, CNRS UMR-S8253, Universite Paris Cite, Institut Necker Enfants Malades, Paris, France; 2 Department of Immunology and Immunotherapy, School of Infection, Inflammation and Immunology, College of Medicine and Health, University of Birmingham, Birmingham, UK; 3 School of Biochemistry and Immunology, Trinity Biomedical Sciences Institute, Trinity College Dublin, Dublin, Ireland

**Keywords:** stable isotope tracing, itaconate metabolism, succinate dehydrogenase

## Abstract

The discovery of itaconate as an immunoregulatory metabolite has transformed the field of immunometabolism and opened multiple therapeutic avenues over the past decade. While the immunological functions of itaconic acid have been extensively studied, several aspects of its biochemistry—particularly in vivo utilization pathways—have remained unclear. In a recent study published in *Nature Metabolism*, Willenbockel et al apply carbon tracing to uncover the metabolic fate of itaconate within the organism. Insights from this work have important implications for understanding the physiological roles of itaconate and for advancing itaconate-based therapeutic strategies.

Since the rediscovery of itaconate as an immunometabolite in 2011 ^[1]^, much work has been done to understand the biochemical and immunological pathways involving this metabolite. Early on, itaconate was shown to inhibit succinate dehydrogenase (SDH), leading to suppression of the tricarboxylic acid cycle (TCA) and oxidative phosphorylation (OXPHOS) ^[2]^. Itaconyl-CoA has later been identified as an inhibitor of methylmalonyl-CoA mutase (MUT) and the erythroid-specific enzyme 5-aminolevulinate synthase, thereby indirectly influencing branched-chain amino acid (BCAA) and fatty acid metabolism ^[3,4]^, as well as heme biosynthesis ^[5]^. Additionally, itaconate can covalently modify cysteine residues via a Michael addition mechanism, impacting various signaling and metabolic pathways, including but not limited to Nuclear factor erythroid 2-related factor 2 (Nrf2), Nuclear factor kappa B (NF-κB), and glycolysis ^[6–8]^. However, the metabolic effects of itaconate are highly context-dependent, varying with cell type and physiological conditions. For instance, in hepatocytes, itaconate paradoxically enhances glycolysis, oxidative phosphorylation, and fatty acid β-oxidation ^[9]^. Given these complexities, understanding how itaconate shapes metabolic activity, particularly in vivo, remains an important objective.

The metabolic pathway of itaconate utilization has been gradually pieced together since the late 1950s, when its activation to itaconyl-CoA in mammals was first demonstrated, followed by hydration to citramalyl-CoA and cleavage into pyruvate and acetyl-CoA ^[10,11]^. Since then, key enzymes involved in this process have been identified, including SUGCT (succinyl-CoA:glutarate-CoA transferase) ^[4]^, and citrate lyase beta-like protein (CLYBL), which was functionally characterized as a citramalyl-CoA lyase ^[3]^. Itaconate can also isomerize to mesaconate, a metabolite with its own regulatory activity ^[12]^. To link together those primary observations and get an insight into tissue distribution of itaconate-mediated metabolic effects in vivo, a recent study by Willenbockel et al ^[13]^ applied pulse administration and carbon tracing of itaconate in rodents.

In response to itaconate injection, the authors reported rapid and dynamic changes in the plasma metabolome, with the levels of itaconate, mesaconate, citramalate, and succinate displaying matching dynamic profiles. They also noted a progressive increase in plasma levels of methylmalonate and BCAA. Taken together, these findings support previous observations on itaconate’s impact on SDH and MUT activity ^[2–4]^. ¹³C tracing enabled authors to further follow the metabolic fate of itaconate in vivo. Strikingly, in contrast to what was observed in hepatocarcinoma cell lines, the authors revealed a swift and substantial conversion of itaconate into acetyl-CoA in mouse liver, followed by engagement in the carnitine shuttle and subsequent incorporation into the TCA cycle and amino acids. Organ-level metabolic profiling provided key insights (Figure 1): while itaconate accumulated prominently in the heart, lungs, kidneys, and liver, its metabolic impact varied across tissues. Based on SDH activity (succinate/fumarate ratio), the proportion of itaconate-derived citrate, and the levels of its derivatives, mesaconate and citramalate, the authors showed that itaconate is actively metabolized in the liver and kidneys, while simultaneously suppressing SDH in those tissues.

**Figure 1.**
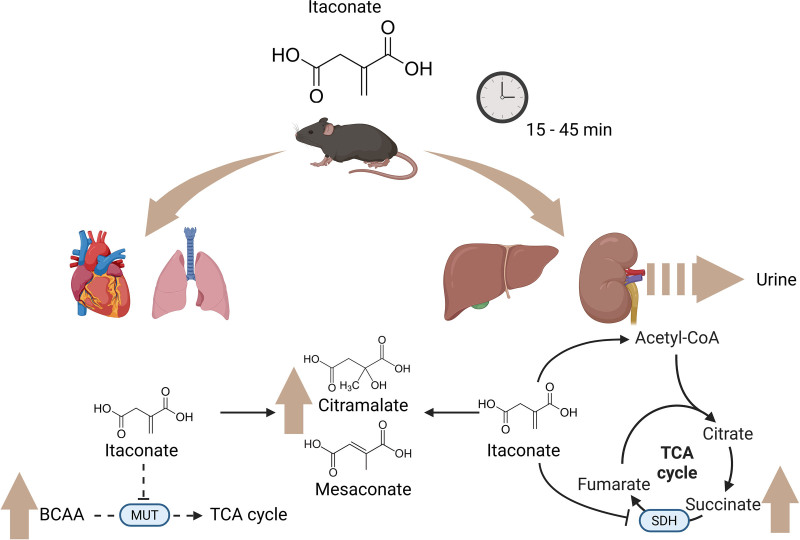
**Following injection in mice, itaconate is rapidly accumulated predominantly in the heart, lungs, kidneys, and liver, with a significant portion excreted in the urine shortly thereafter.** In these organs, itaconate is metabolized into mesaconate and citramalate. Notably, itaconate strongly suppresses succinate dehydrogenase (SDH) activity along with active incorporation into the tricarboxylic acid (TCA) cycle in the liver and the kidney. Those pathways were much less affected in the heart and lungs. Instead, itaconate induces the accumulation of branched-chain amino acids (BCAA) in these tissues due to itaconyl-CoA-mediated inhibition of methylmalonyl-CoA mutase (MUT) (dashed lines denote simplified pathways). Created in BioRender. Nosenko, M. (2025) https://BioRender.com/b60wy51.

Itaconate is produced and accumulated systemically upon various conditions, including infections ^[14]^, autoimmunity ^[15]^, neurological ^[16]^ and metabolic ^[9,17]^ diseases, as well as cancer ^[18]^, substantially contributing to their pathogenesis. While the majority of mechanisms involving itaconate were studied in vitro, they are not always applied in physiological contexts, which highlights the importance of studying itaconate metabolism and functions in vivo. Current work confirms previous observations that inactivation of SDH is the main metabolic effect of itaconate in vivo, and it was shown to be involved in the regulation of oxidative stress in ischemia/reperfusion ^[2,19]^ and production of Interleukin-6 (IL-6) in local ^[20]^ and acute systemic ^[21]^ inflammation. New findings suggest that itaconate could also directly fuel the TCA cycle, potentially contributing to mitochondrial metabolism and OXPHOS. While in mouse liver and kidneys, a proportion of itaconate was rapidly converted into citrate, likely via the generation of acetyl-CoA; this transformation was not observed in cultured cells. Given the involvement of the enzyme CLYBL in this process and its broad but heterogeneous expression across tissues, it is possible that the lack of this metabolic conversion in vitro is due to insufficient representation of CLYBL in the cell lines used.

Another important outcome of itaconate is irreversible inactivation of MUT, affecting the metabolism of BCAA and the availability of vitamin B_12_. BCAA metabolism is essential for the normal physiology of skeletal muscle, adipose tissue, and the liver, and its dysregulation has been linked to metabolic disorders, including obesity and type 2 diabetes ^[22]^. Given the growing body of evidence implicating itaconate in the pathogenesis of these conditions, its potential role in regulating BCAA metabolism warrants further investigation. Finally, a combination of these diverse metabolic and immunoregulatory effects of itaconate, along with enzymatic or enzyme-independent roles of aconitate decarboxylase 1, potentially explains the ambiguous role of this metabolic pathway in obesity ^[23,24]^.

Many open questions in the field of itaconate metabolism require further investigation. First, it is worth noting that the current study was only performed in male animals, so it remains to be determined whether the same pathways and tissue-specificities are at play in females. While the study determines the metabolic fate of itaconate at steady state, it is not clear which pathways are involved in the utilization of endogenously produced itaconic acid upon inflammation. Continuous slow production of itaconate, at steady state or in the context of chronic inflammation, may result in different patterns of metabolism, not reflected in pulse administration studies. For example, it is not clear which cells experience preferential SDH suppression or accumulation of itaconate-derived TCA intermediates. Insights into the distribution and metabolism of itaconate in different organs represent the major advance of this manuscript, and the fate of itaconate in those tissues is yet to be determined. Specifically, tissues with higher uptake of itaconate, such as heart, lungs, and kidneys, are of interest. Interestingly, the authors’ data indicate superior accumulation of itaconate in the heart with no signs of active SDH inhibition. This raises a question of itaconate metabolic fate in cardiomyocytes, especially in light of the previous findings showing that treatment with an itaconate derivative is protective in a myocardial ischemia-reperfusion model ^[2]^. Whether the accumulation of itaconate in the lungs has pro- or anti-inflammatory outcomes might depend on the model and cell types involved ^[20]^. Finally, utilization pathways for itaconate derivatives, such as dimethyl itaconate or 4-octyl itaconate, are yet to be investigated, which has important implications for the development of itaconate-based therapies.

Altogether, the study provides important insights into the metabolic fate of itaconate in vivo, complementing current knowledge about its immunoregulatory and biochemical actions.

## Author contribution

D.E.A. and M.A.N. wrote the original draft and prepared the figure; S.A.C. reviewed and edited the manuscript.

## Conflict of interest

The authors declare that they have no conflicts of interest.

## Funding

D.E.A. is supported by the grant from Horizon Europe (Intercept-T2D, 101095433); S.A.C. is supported by Wellcome Trust (Early Career Award, 300104/Z/23/Z); M.A.N. is funded by the European Union (Marie Skłodowska-Curie Postdoctoral Fellowship, TANK_Sepsis, grant agreement No 101111015).
